# Synthesis and crystal structure of deca­carbon­yl(μ_3_-3,7-di­thia­nonane-1,9-di­thiol­ato)bis­(μ_2_-propane-1,3-di­thiol­ato)nickel(II)tetra­iron(II) di­chloro­methane disolvate

**DOI:** 10.1107/S2056989018001731

**Published:** 2018-02-13

**Authors:** Gan Ren, Ge Sang

**Affiliations:** a Science and Technology on Surface Physics and Chemistry Laboratory, Jiangyou 621908, People’s Republic of China; b Institute of Materials, China Academy of Engineering Physics, Jiangyou 621908, People’s Republic of China

**Keywords:** crystal structure, FeS, catalysis, proton reduction, electrochemistry, H-cluster, [FeFe]-hydrogenase, hydrogen bonding

## Abstract

The title compound,, [Fe_4_Ni(C_3_H_6_S_2_)_2_(C_7_H_14_S_4_)(CO)_10_]·2CH_2_Cl_2_, is reported as a biomimic model for the active site of [FeFe]-hydrogenases. Bis(2-mercaptoeth­yl)-1,3-propane­dithio ether nickel(II) was firstly introduced into [Fe_2_(C_3_H_6_S_2_)(CO)_5_] as an S-containing ligand. It coordinates with two [Fe_2_(C_3_H_6_S_2_)(CO)_5_] groups, and a five-metal core complex is formed. The Fe_2_S_2_ core is in a butterfly conformation. The Fe—Fe distances in the [Fe_2_(C_3_H_6_S_2_)(CO)_5_] groups are 2.5126 (6) and 2.5086 (7) Å. The distances between the adjacent Fe and Ni atoms are 3.5322 (1) and 3.5143 (1) Å. There are intra­molecular C—H⋯O and C—H⋯S contacts present in the complex. In the crystal, the five metal cores are linked *via* C—H⋯O hydrogen bonds, forming columns lying parallel to (110). The di­chloro­methane solvent mol­ecules are each partially disordered over two positions and only one is linked to the five-metal core complex by a C—H⋯O hydrogen bond.

## Abstract 2

Synthesis and structural studies of a Ni^II^S_4_–2{2Fe2S}model for the H-cluster of [FeFe]-hydrogenase.

## Chemical context   

[FeFe]-hydrogenases are special enzymes in numerous microorganisms, which catalyse hydrogen evolution or splitting. Crystallographic and IR spectroscopic studies on [FeFe]-hydrogenases have revealed that the active site of [FeFe]-hydrogenases is comprised of a 2Fe2S butterfly structure containing diatomic ligands CO and CN^−^, a cysteinyl-S ligand connecting to a 4Fe4S subcluster, and a three-atom linker bridged between the two S atoms of the Fe_2_S_2_ H-cluster (Tard *et al.*, 2005 ▸; Tard & Pickett 2009 ▸).
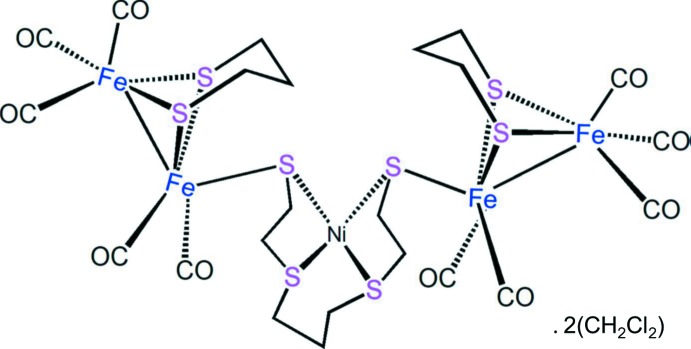


Vigorous functional modelling studies have commenced with the [2Fe2S] subunit, but less attention has been paid in structural modelling studies to the overall H-cluster. The 4Fe4S sub-cluster was found to work as electrons relaying in numerous microorganisms. The 4Fe4S sub-cluster itself is a strong electron-donating group. The limited number of studies on the 4Fe4S sub-cluster encouraged us to synthesize the title compound, introducing bis­(2-mercaptoeth­yl)-1,3-propane­dithio­ethernickel(II) into the 2Fe2S cluster to mimic the 4Fe4S sub-cluster.

## Structural commentary   

**Figure 1:**
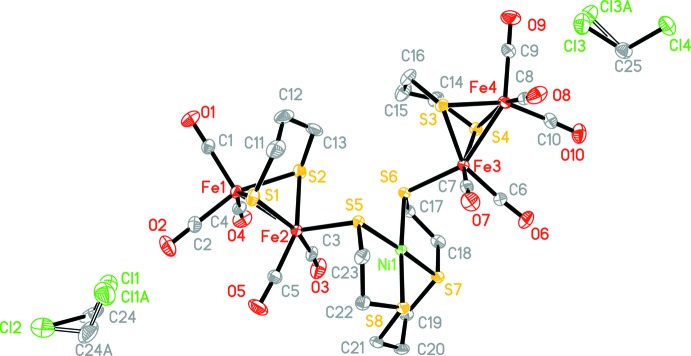
The mol­ecular structure of the title compound, with the atom labelling. Displacement ellipsoids are drawn at the 30% probability level. H atoms have been omitted for clarity, and the disordered atoms of the CH_2_Cl_2_ mol­ecules are shown with suffix A.

The structure of the title compound, illustrated in Fig. 1 ▸, resembles the active site of [FeFe]-hydrogenases, with two butterfly architectonic 2Fe2S clusters and one planar NiS4 core. The Ni atom is displaced by only 0.0023 Å (5) out of the mean plane of the four S atoms. The Fe1—Fe2 and Fe3—Fe4 bond lengths are 2.5126 (6) and 2.5086 (7) Å, respectively, slightly shorter than those in the structures of natural enzymes (*ca* 2.6 Å: Peters *et al.*, 1998 ▸; Nicolet *et al.*, 1999 ▸). The Fe2⋯Ni1 and Fe3⋯Ni1 distances are 3.5320 (6) and 3.5144 (6) Å, respectively. There are intra­molecular C—H⋯O and C—H⋯S contacts present in the complex (Table 1 ▸).

The introduction of bis­(2-mercaptoeth­yl)-1,3-propane­dithio­ethernickel(II) into the 2Fe2S cluster results in a significant red shift for the C=O group in the IR spectrum; the highest and lowest absorption wave-numbers differ by 123 cm^−1^, which suggests a significant difference in the electron density between the two Fe^II^ ions. The IR signal therefore indicates that bis­(2-mercaptoeth­yl)-1,3-propane­dithio­ether­nickel(II) can mimic the strong electron-donating ability of the 4Fe4S subcluster.

## Superamolecular features   

**Figure 2:**
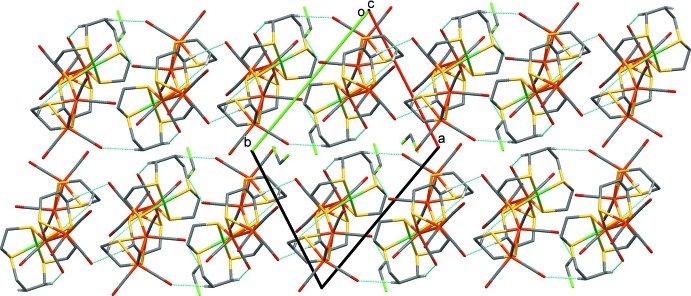
A view along the *c* axis of the crystal packing of the title compound. The hydrogen bonds are shown as dashed lines (see Table 1 ▸), and H atoms not involved in these inter­actions have been omitted.

In the crystal, the five-metal core complexes are linked *via* C—H⋯O hydrogen bonds, forming columns propagating along [110]; see Table 1 ▸ and Fig. 2 ▸. The di­chloro­methane solvent mol­ecules are each partially disordered over two positions and only one is linked to the five-metal core complex by a C—H⋯O hydrogen bond (Fig. 2 ▸, Table 1 ▸).

## Database survey   

A search of the Cambridge Structural Database (Version 5.38, update May 2017; Groom *et al.*, 2016 ▸) gave over 100 hits for the μ–propane­dithiol­ate diiron penta­carbonyl skeleton. Examining these structures, it can be seen that during the past few years, a series of model complexes [(μ-pdt)Fe_2_(CO)_5_ *L*] (*L* = PMe_3_, PPh_3_, ect., pdt = propane­dithiol­ate) were synthesized as H-cluster analogues of [FeFe]-hydrogenase (Dong *et al.*, 2006 ▸; Felton *et al.*, 2009 ▸). However, less attention has been paid to structural modelling studies to the overall H-cluster. Pickett and coworkers have synthesized and spectroscopically characterized [6Fe6S] model complexes, but without crystal structure analyses (Tard *et al.*, 2005 ▸). Other model complexes reported as analogues of [2Fe3S] or [3Fe3S] subunits have been reported (Tard *et al.*, 2009 ▸). A novel 2Fe2S–Fe^II^ model complex *A* and its analogues [(μ-pdt)Fe_2_(CO)_5_]_2_ *M*(sip)_2_ [*M* = Fe (A), Ni (*B*); pdt = propane­dithiol­ate; sip = sulfanyl­propyl­imino­methyl­pyridine] have been reported (CSD refcodes ALIZIF and ALIZOL, respectively; Hu *et al.*, 2010 ▸).

## Experimental   

All reactions and operations were carried out under a dry, pre-purified nitro­gen atmosphere with standard Schlenk techniques. All solvents were dried and distilled prior to use according to standard methods. The starting materials, Fe_2_(C_3_H_6_S_2_)(CO)_6_ (*A*) and NiC_7_H_14_S_4_ (*B*), were prepared according to literature methods (Maiolo *et al.*, 1981 ▸). Me_3_NO·2H_2_O (1 mmol, 0.111 g) was added to a CH_3_CN solution of complex *A* (1 mmol, 0.168 g) under an N_2_ atmos­phere with stirring. A CH_2_Cl_2_/CH_3_OH (2:1) solution of complex B (0.5 mmol, 0.143 g) was added after 30 min. The colour of the solution changed gradually from red to dark red. After one h, the solvent was removed under reduced pressure. The residue was purified by column chromatography on silica gel using hexane as eluent to give the title compound as a red solid (yield 0.164 g, 51%). It is unstable in solution in air. Single crystals suitable for the X-ray diffraction study were obtained by slow evaporation of a solution in CH_2_Cl_2_/hexane (1:10, *v*/*v*) at 263 K. IR (CH_2_Cl_2_, cm^−1^): *ν* (CO) 2020 (*m*), 1956 (*s*), 1897 (*v*). Analysis calculated for C_25_H_30_Cl_4_Fe_4_NiO_10_S_8_: C, 25.64; H, 2.58; S, 21.91%; Found: C, 25.62; H, 2.56; S, 21.92%.

## Refinement   

Crystal data, data collection and structure refinement details are summarized in Table 2 ▸. All hydrogen atoms were placed in calculated positions and refined as riding: C—H = 0.97 Å with *U*_iso_(H) = 1.2*U*_eq_(C). The di­chloro­methane solvent mol­ecules are each partially disordered over two positions. That involving atoms Cl1 and Cl2 have atoms Cl1/Cl1*A* and C24/C24*A* with fixed occupancies of 0.5 each, while that involving atoms Cl3 and Cl4 have atom Cl3/Cl3*A* with a refined occupancy ratio of 0.77 (6):0.23 (6).

## Supplementary Material

Crystal structure: contains datablock(s) I, Global. DOI: 10.1107/S2056989018001731/su5420sup1.cif

Structure factors: contains datablock(s) I. DOI: 10.1107/S2056989018001731/su5420Isup2.hkl

CCDC references: 1424813, 1821349

Additional supporting information:  crystallographic information; 3D view; checkCIF report

## Figures and Tables

**Table 1:** Hydrogen-bond geometry (Å, °)

*D*—H⋯*A*	*D*—H	H⋯*A*	*D*⋯*A*	*D*—H⋯*A*
C19—H19*A*⋯O3	0.97	2.52	3.440 (5)	159
C15—H15*B*⋯S6	0.97	2.83	3.578 (4)	134
C13—H13*B*⋯O4^i^	0.97	2.57	3.470 (5)	154
C20—H20*A*⋯O8^ii^	0.97	2.57	3.263 (5)	128
C24—H24*A*⋯O7^iii^	0.97	2.44	3.08 (2)	123

Symmetry codes: (i) 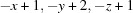; (ii) 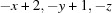; (iii) 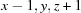.

**Table 2:** Experimental details

Crystal data	
Chemical formula	[Fe_4_Ni(C_3_H_6_S_2_)_2_(C_7_H_14_S_4_)(CO)_10_]·2CH_2_Cl_2_
*M*_r_	1170.88
Crystal system, space group	Triclinic, *P* 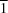
Temperature (K)	243
*a*, *b*, *c* (Å)	12.4340 (8), 13.9981 (10), 14.4912 (9)
α, β, γ (°)	77.860 (4), 67.570 (4), 64.036 (4)
*V* (Å^3^)	2093.3 (2)
*Z*	2
Radiation type	Mo *K*α
μ (mm^−1^)	2.50
Crystal size (mm)	0.40 × 0.30 × 0.20

Data collection	
Diffractometer	Bruker APEXII area detector
Absorption correction	Multi-scan (*SADABS*; Bruker, 2007 ▸)
*T*_min_, *T*_max_	0.435, 0.635
No. of measured, independent and observed [*I* > 2σ(*I*)] reflections	20488, 7352, 5928
*R*_int_	0.034
(sin θ/λ)_max_ (Å^−1^)	0.595

Refinement	
*R*[*F* ^2^ > 2σ(*F* ^2^)], *wR*(*F* ^2^), *S*	0.032, 0.075, 1.01
No. of reflections	7352
No. of parameters	497
No. of restraints	12
H-atom treatment	H-atom parameters constrained
Δρ_max_, Δρ_min_ (e Å^−3^)	0.94, −0.59

Computer programs: *APEX2* and *SAINT-Plus* (Bruker, 2007 ▸), *SHELXS97*, *SHELXL97* and *SHELXTL* (Sheldrick, 2008 ▸), *Mercury*(Macrae *et al.*, 2008 ▸), *PLATON* (Spek, 2009 ▸) and *publCIF* (Westrip, 2010 ▸).

## supplementary crystallographic information

### Crystal data

**Table:**

[Fe_4_Ni(C_3_H_6_S_2_)_2_(C_7_H_14_S_4_)(CO)_10_]·2CH_2_Cl_2_	*Z* = 2
*M**_r_* = 1170.88	*F*(000) = 1176
Triclinic, *P*1	*D*_x_ = 1.858 Mg m^−^^3^
Hall symbol: -P 1	Mo *K*α radiation, λ = 0.71073 Å
*a* = 12.4340 (8) Å	Cell parameters from 6326 reflections
*b* = 13.9981 (10) Å	θ = 1.9–25.0°
*c* = 14.4912 (9) Å	µ = 2.50 mm^−^^1^
α = 77.860 (4)°	*T* = 243 K
β = 67.570 (4)°	Block, red
γ = 64.036 (4)°	0.40 × 0.30 × 0.20 mm
*V* = 2093.3 (2) Å^3^	

### Data collection

**Table:**

Bruker APEXII area detector diffractometer	7352 independent reflections
Radiation source: fine-focus sealed tube	5928 reflections with *I* > 2σ(*I*)
Graphite monochromator	*R*_int_ = 0.034
phi and ω scans	θ_max_ = 25.0°, θ_min_ = 1.9°
Absorption correction: multi-scan (SADABS; Bruker, 2007)	*h* = −14→14
*T*_min_ = 0.435, *T*_max_ = 0.635	*k* = −16→16
20488 measured reflections	*l* = −17→17

### Refinement

**Table:**

Refinement on *F*^2^	Primary atom site location: structure-invariant direct methods
Least-squares matrix: full	Secondary atom site location: difference Fourier map
*R*[*F*^2^ > 2σ(*F*^2^)] = 0.032	Hydrogen site location: inferred from neighbouring sites
*wR*(*F*^2^) = 0.075	H-atom parameters constrained
*S* = 1.01	*w* = 1/[σ^2^(*F*_o_^2^) + (0.0322*P*)^2^ + 1.0504*P*] where *P* = (*F*_o_^2^ + 2*F*_c_^2^)/3
7352 reflections	(Δ/σ)_max_ = 0.002
497 parameters	Δρ_max_ = 0.94 e Å^−^^3^
12 restraints	Δρ_min_ = −0.59 e Å^−^^3^

### Special details

**Table:**

Geometry. All esds (except the esd in the dihedral angle between two l.s. planes) are estimated using the full covariance matrix. The cell esds are taken into account individually in the estimation of esds in distances, angles and torsion angles; correlations between esds in cell parameters are only used when they are defined by crystal symmetry. An approximate (isotropic) treatment of cell esds is used for estimating esds involving l.s. planes.
Refinement. Refinement of F^2^ against ALL reflections. The weighted R-factor wR and goodness of fit S are based on F^2^, conventional R-factors R are based on F, with F set to zero for negative F^2^. The threshold expression of F^2^ > 2sigma(F^2^) is used only for calculating R-factors(gt) etc. and is not relevant to the choice of reflections for refinement. R-factors based on F^2^ are statistically about twice as large as those based on F, and R- factors based on ALL data will be even larger.

### Fractional atomic coordinates and isotropic or equivalent isotropic displacement parameters (Å^2^)

**Table:**

	*x*	*y*	*z*	*U*_iso_*/*U*_eq_	Occ. (<1)
Fe1	0.63313 (4)	0.79098 (4)	0.59106 (3)	0.02955 (12)	
Fe2	0.71720 (4)	0.66176 (3)	0.45877 (3)	0.02572 (11)	
Fe3	0.81044 (4)	0.79149 (3)	0.02362 (3)	0.02590 (11)	
Fe4	0.88790 (4)	0.91380 (4)	−0.11165 (4)	0.03041 (12)	
Ni1	0.75218 (3)	0.60567 (3)	0.21998 (3)	0.02502 (10)	
S1	0.84093 (8)	0.68240 (7)	0.52818 (7)	0.0376 (2)	
S2	0.64699 (8)	0.84026 (7)	0.42929 (7)	0.0373 (2)	
S3	0.90803 (7)	0.88819 (7)	0.04133 (7)	0.0320 (2)	
S4	0.67953 (7)	0.94975 (7)	−0.02773 (6)	0.03083 (19)	
S5	0.85931 (7)	0.62317 (6)	0.29938 (6)	0.02740 (18)	
S6	0.68591 (7)	0.77643 (6)	0.18533 (6)	0.02789 (18)	
S7	0.62939 (8)	0.59262 (7)	0.15145 (6)	0.0325 (2)	
S8	0.83260 (8)	0.43272 (6)	0.24203 (6)	0.03208 (19)	
O1	0.6078 (2)	0.9814 (2)	0.6672 (2)	0.0544 (7)	
O2	0.6228 (3)	0.6580 (3)	0.7763 (2)	0.0759 (10)	
O3	0.4837 (2)	0.6692 (2)	0.4504 (2)	0.0603 (8)	
O4	0.3661 (2)	0.8440 (2)	0.6263 (2)	0.0582 (8)	
O5	0.7473 (3)	0.4637 (2)	0.5800 (2)	0.0745 (10)	
O6	0.7703 (3)	0.6795 (2)	−0.0997 (2)	0.0656 (8)	
O7	1.0350 (2)	0.5987 (2)	0.0248 (2)	0.0556 (7)	
O8	1.1618 (2)	0.7912 (2)	−0.1818 (2)	0.0583 (8)	
O9	0.8921 (3)	1.1258 (2)	−0.1749 (2)	0.0697 (9)	
O10	0.8462 (3)	0.8648 (2)	−0.2789 (2)	0.0589 (8)	
C1	0.6208 (3)	0.9071 (3)	0.6366 (3)	0.0370 (8)	
C2	0.6287 (4)	0.7096 (3)	0.7035 (3)	0.0453 (9)	
C3	0.5787 (3)	0.6654 (3)	0.4492 (3)	0.0376 (8)	
C4	0.4700 (3)	0.8252 (3)	0.6137 (3)	0.0396 (9)	
C5	0.7381 (3)	0.5402 (3)	0.5288 (3)	0.0418 (9)	
C6	0.7805 (3)	0.7242 (3)	−0.0477 (3)	0.0387 (8)	
C7	0.9462 (3)	0.6750 (3)	0.0261 (3)	0.0359 (8)	
C8	1.0549 (3)	0.8382 (3)	−0.1562 (3)	0.0383 (8)	
C9	0.8892 (3)	1.0439 (3)	−0.1504 (3)	0.0422 (9)	
C10	0.8630 (3)	0.8853 (3)	−0.2144 (3)	0.0408 (9)	
C11	0.9377 (4)	0.7519 (4)	0.4431 (3)	0.0619 (13)	
H11A	0.9970	0.7488	0.4729	0.074*	
H11B	0.9867	0.7140	0.3819	0.074*	
C12	0.8670 (4)	0.8653 (4)	0.4168 (3)	0.0689 (15)	
H12A	0.9276	0.8961	0.3757	0.083*	
H12B	0.8182	0.9036	0.4777	0.083*	
C13	0.7789 (4)	0.8813 (3)	0.3618 (3)	0.0578 (12)	
H13A	0.8285	0.8426	0.3011	0.069*	
H13B	0.7442	0.9562	0.3419	0.069*	
C14	0.6123 (3)	1.0571 (3)	0.0566 (3)	0.0460 (9)	
H14A	0.6164	1.1208	0.0158	0.055*	
H14B	0.5233	1.0706	0.0901	0.055*	
C15	0.6679 (4)	1.0435 (3)	0.1332 (3)	0.0607 (11)	
H15A	0.6275	1.1110	0.1645	0.073*	
H15B	0.6428	0.9928	0.1839	0.073*	
C16	0.8032 (3)	1.0094 (3)	0.1091 (3)	0.0498 (10)	
H16A	0.8201	0.9999	0.1712	0.060*	
H16B	0.8265	1.0669	0.0700	0.060*	
C17	0.5253 (3)	0.8050 (3)	0.1922 (3)	0.0358 (8)	
H17A	0.4742	0.8006	0.2616	0.043*	
H17B	0.4885	0.8772	0.1659	0.043*	
C18	0.5215 (3)	0.7290 (3)	0.1343 (3)	0.0389 (9)	
H18A	0.5443	0.7507	0.0637	0.047*	
H18B	0.4359	0.7323	0.1563	0.047*	
C19	0.5269 (3)	0.5391 (3)	0.2543 (3)	0.0384 (8)	
H19A	0.4915	0.5806	0.3124	0.046*	
H19B	0.4574	0.5444	0.2356	0.046*	
C20	0.6004 (3)	0.4242 (3)	0.2799 (3)	0.0406 (9)	
H20A	0.6433	0.3859	0.2190	0.049*	
H20B	0.5402	0.3944	0.3244	0.049*	
C21	0.6982 (3)	0.4026 (3)	0.3286 (3)	0.0373 (8)	
H21A	0.7284	0.3284	0.3518	0.045*	
H21B	0.6586	0.4456	0.3862	0.045*	
C22	0.9260 (3)	0.4059 (3)	0.3229 (3)	0.0390 (9)	
H22A	0.8729	0.4082	0.3925	0.047*	
H22B	0.9939	0.3356	0.3121	0.047*	
C23	0.9805 (3)	0.4890 (3)	0.2984 (3)	0.0380 (9)	
H23A	1.0450	0.4776	0.2329	0.046*	
H23B	1.0204	0.4815	0.3469	0.046*	
C24	0.2347 (12)	0.6444 (8)	1.0636 (13)	0.084 (5)	0.50
H24A	0.2297	0.5877	1.0390	0.101*	0.50
H24B	0.1599	0.7081	1.0624	0.101*	0.50
Cl1	0.3634 (8)	0.6661 (6)	0.9840 (8)	0.099 (2)	0.50
C24A	0.2888 (15)	0.5782 (14)	1.0674 (11)	0.111 (5)	0.50
H24C	0.2191	0.6039	1.0415	0.133*	0.50
H24D	0.3270	0.5013	1.0658	0.133*	0.50
Cl1A	0.4044 (7)	0.6293 (7)	0.9861 (7)	0.106 (2)	0.50
Cl2	0.23047 (12)	0.61093 (11)	1.18636 (13)	0.0906 (4)	
C25	0.7905 (4)	1.1147 (4)	−0.4497 (3)	0.0579 (11)	
H25A	0.7078	1.1437	−0.4576	0.069*	
H25B	0.8105	1.0404	−0.4274	0.069*	
Cl3	0.7839 (13)	1.1824 (16)	−0.3584 (7)	0.077 (2)	0.77 (6)
Cl3A	0.7681 (19)	1.215 (3)	−0.382 (4)	0.070 (7)	0.23 (6)
Cl4	0.90311 (11)	1.12301 (10)	−0.56564 (9)	0.0686 (3)	

### Atomic displacement parameters (Å^2^)

**Table:**

	*U*^11^	*U*^22^	*U*^33^	*U*^12^	*U*^13^	*U*^23^
Fe1	0.0318 (2)	0.0291 (3)	0.0275 (3)	−0.0109 (2)	−0.0094 (2)	−0.0046 (2)
Fe2	0.0280 (2)	0.0249 (2)	0.0249 (3)	−0.01072 (19)	−0.00932 (19)	−0.00100 (19)
Fe3	0.0287 (2)	0.0246 (2)	0.0253 (3)	−0.01209 (19)	−0.00882 (19)	0.00017 (19)
Fe4	0.0321 (2)	0.0322 (3)	0.0283 (3)	−0.0166 (2)	−0.0092 (2)	0.0031 (2)
Ni1	0.0291 (2)	0.0233 (2)	0.0248 (2)	−0.01258 (17)	−0.00913 (17)	0.00011 (17)
S1	0.0312 (4)	0.0456 (5)	0.0374 (5)	−0.0104 (4)	−0.0159 (4)	−0.0070 (4)
S2	0.0453 (5)	0.0255 (5)	0.0313 (5)	−0.0049 (4)	−0.0147 (4)	0.0011 (4)
S3	0.0328 (4)	0.0336 (5)	0.0357 (5)	−0.0150 (4)	−0.0157 (4)	−0.0015 (4)
S4	0.0295 (4)	0.0324 (5)	0.0315 (5)	−0.0116 (3)	−0.0138 (4)	0.0020 (4)
S5	0.0296 (4)	0.0275 (4)	0.0267 (4)	−0.0118 (3)	−0.0101 (3)	−0.0017 (3)
S6	0.0342 (4)	0.0249 (4)	0.0256 (4)	−0.0141 (3)	−0.0098 (3)	0.0021 (3)
S7	0.0377 (4)	0.0347 (5)	0.0326 (5)	−0.0199 (4)	−0.0139 (4)	0.0008 (4)
S8	0.0383 (4)	0.0248 (4)	0.0320 (5)	−0.0123 (4)	−0.0096 (4)	−0.0033 (4)
O1	0.0494 (15)	0.0471 (17)	0.072 (2)	−0.0168 (13)	−0.0161 (14)	−0.0250 (15)
O2	0.101 (2)	0.079 (2)	0.046 (2)	−0.038 (2)	−0.0312 (19)	0.0208 (17)
O3	0.0382 (14)	0.097 (2)	0.0568 (19)	−0.0337 (15)	−0.0072 (13)	−0.0268 (17)
O4	0.0315 (14)	0.0600 (19)	0.079 (2)	−0.0138 (13)	−0.0101 (13)	−0.0212 (16)
O5	0.108 (2)	0.0373 (17)	0.0499 (19)	−0.0269 (17)	−0.0060 (18)	0.0124 (14)
O6	0.092 (2)	0.075 (2)	0.0529 (19)	−0.0474 (18)	−0.0224 (17)	−0.0175 (16)
O7	0.0447 (15)	0.0417 (17)	0.0584 (19)	−0.0022 (13)	−0.0121 (14)	−0.0021 (14)
O8	0.0347 (15)	0.0520 (17)	0.083 (2)	−0.0141 (13)	−0.0042 (14)	−0.0303 (16)
O9	0.105 (2)	0.0460 (19)	0.072 (2)	−0.0437 (18)	−0.0391 (19)	0.0189 (16)
O10	0.0622 (17)	0.080 (2)	0.0408 (17)	−0.0291 (16)	−0.0211 (14)	−0.0068 (15)
C1	0.0333 (18)	0.041 (2)	0.036 (2)	−0.0129 (16)	−0.0103 (16)	−0.0072 (17)
C2	0.050 (2)	0.050 (2)	0.036 (2)	−0.0194 (19)	−0.0140 (19)	−0.005 (2)
C3	0.0376 (19)	0.046 (2)	0.030 (2)	−0.0165 (17)	−0.0061 (16)	−0.0129 (17)
C4	0.041 (2)	0.034 (2)	0.042 (2)	−0.0118 (16)	−0.0118 (17)	−0.0096 (17)
C5	0.050 (2)	0.032 (2)	0.033 (2)	−0.0151 (17)	−0.0038 (17)	−0.0047 (17)
C6	0.048 (2)	0.038 (2)	0.034 (2)	−0.0223 (17)	−0.0109 (17)	−0.0021 (17)
C7	0.0370 (19)	0.037 (2)	0.030 (2)	−0.0158 (17)	−0.0059 (16)	−0.0019 (16)
C8	0.044 (2)	0.041 (2)	0.039 (2)	−0.0256 (18)	−0.0107 (17)	−0.0049 (17)
C9	0.050 (2)	0.043 (2)	0.036 (2)	−0.0220 (19)	−0.0148 (18)	0.0044 (18)
C10	0.0358 (19)	0.050 (2)	0.034 (2)	−0.0178 (17)	−0.0104 (17)	0.0029 (18)
C11	0.047 (2)	0.087 (4)	0.065 (3)	−0.041 (2)	−0.005 (2)	−0.022 (3)
C12	0.073 (3)	0.077 (3)	0.064 (3)	−0.060 (3)	0.020 (2)	−0.031 (3)
C13	0.082 (3)	0.032 (2)	0.045 (3)	−0.031 (2)	0.003 (2)	0.0018 (18)
C14	0.0418 (18)	0.0329 (19)	0.056 (2)	−0.0040 (15)	−0.0186 (17)	−0.0082 (17)
C15	0.058 (2)	0.053 (2)	0.067 (2)	−0.0070 (18)	−0.0216 (19)	−0.0259 (19)
C16	0.055 (2)	0.045 (2)	0.057 (2)	−0.0145 (17)	−0.0242 (19)	−0.0190 (19)
C17	0.0304 (17)	0.036 (2)	0.036 (2)	−0.0127 (15)	−0.0085 (15)	0.0046 (16)
C18	0.0354 (18)	0.044 (2)	0.043 (2)	−0.0199 (16)	−0.0191 (17)	0.0078 (17)
C19	0.0392 (19)	0.042 (2)	0.041 (2)	−0.0240 (17)	−0.0126 (17)	0.0006 (17)
C20	0.046 (2)	0.035 (2)	0.045 (2)	−0.0268 (17)	−0.0076 (18)	−0.0004 (17)
C21	0.047 (2)	0.0281 (19)	0.034 (2)	−0.0196 (16)	−0.0077 (17)	0.0048 (15)
C22	0.0445 (19)	0.0256 (19)	0.041 (2)	−0.0052 (16)	−0.0180 (17)	−0.0022 (16)
C23	0.0303 (17)	0.035 (2)	0.044 (2)	−0.0039 (15)	−0.0128 (16)	−0.0100 (17)
C24	0.079 (8)	0.035 (6)	0.177 (14)	−0.002 (5)	−0.098 (9)	−0.021 (7)
Cl1	0.130 (5)	0.091 (4)	0.092 (3)	−0.040 (3)	−0.057 (3)	−0.006 (3)
C24A	0.139 (14)	0.157 (15)	0.095 (10)	−0.107 (12)	−0.033 (10)	−0.025 (12)
Cl1A	0.114 (5)	0.147 (7)	0.088 (3)	−0.087 (5)	−0.039 (3)	0.021 (4)
Cl2	0.0710 (8)	0.0752 (9)	0.1187 (13)	−0.0267 (7)	−0.0299 (8)	0.0007 (8)
C25	0.050 (2)	0.060 (3)	0.061 (3)	−0.026 (2)	−0.012 (2)	−0.002 (2)
Cl3	0.052 (3)	0.130 (5)	0.061 (2)	−0.049 (3)	−0.0078 (15)	−0.021 (3)
Cl3A	0.041 (4)	0.086 (12)	0.090 (14)	−0.015 (6)	−0.021 (6)	−0.038 (8)
Cl4	0.0661 (7)	0.0681 (8)	0.0531 (7)	−0.0241 (6)	−0.0090 (6)	0.0070 (6)

### Geometric parameters (Å, º)

**Table:**

Fe1—C4	1.778 (4)	C11—H11A	0.9700
Fe1—C2	1.779 (4)	C11—H11B	0.9700
Fe1—C1	1.805 (4)	C12—C13	1.504 (6)
Fe1—S2	2.2620 (10)	C12—H12A	0.9700
Fe1—S1	2.2724 (9)	C12—H12B	0.9700
Fe1—Fe2	2.5126 (6)	C13—H13A	0.9700
Fe2—C5	1.755 (4)	C13—H13B	0.9700
Fe2—C3	1.759 (3)	C14—C15	1.460 (5)
Fe2—S2	2.2613 (10)	C14—H14A	0.9700
Fe2—S1	2.2703 (9)	C14—H14B	0.9700
Fe2—S5	2.3073 (9)	C15—C16	1.449 (5)
Fe3—C6	1.761 (4)	C15—H15A	0.9700
Fe3—C7	1.767 (4)	C15—H15B	0.9700
Fe3—S4	2.2769 (9)	C16—H16A	0.9700
Fe3—S3	2.2787 (9)	C16—H16B	0.9700
Fe3—S6	2.2897 (9)	C17—C18	1.513 (5)
Fe3—Fe4	2.5086 (7)	C17—H17A	0.9700
Fe4—C10	1.783 (4)	C17—H17B	0.9700
Fe4—C8	1.786 (4)	C18—H18A	0.9700
Fe4—C9	1.794 (4)	C18—H18B	0.9700
Fe4—S3	2.2619 (10)	C19—C20	1.510 (5)
Fe4—S4	2.2708 (9)	C19—H19A	0.9700
Ni1—S6	2.1750 (9)	C19—H19B	0.9700
Ni1—S5	2.1754 (9)	C20—C21	1.520 (5)
Ni1—S8	2.1822 (9)	C20—H20A	0.9700
Ni1—S7	2.1974 (9)	C20—H20B	0.9700
S1—C11	1.817 (4)	C21—H21A	0.9700
S2—C13	1.828 (4)	C21—H21B	0.9700
S3—C16	1.825 (3)	C22—C23	1.507 (5)
S4—C14	1.828 (4)	C22—H22A	0.9700
S5—C23	1.820 (3)	C22—H22B	0.9700
S6—C17	1.823 (3)	C23—H23A	0.9700
S7—C18	1.822 (3)	C23—H23B	0.9700
S7—C19	1.823 (3)	C24—Cl1	1.689 (19)
S8—C22	1.826 (4)	C24—Cl2	1.728 (16)
S8—C21	1.827 (3)	C24—H24A	0.9700
O1—C1	1.139 (4)	C24—H24B	0.9700
O2—C2	1.142 (5)	C24A—Cl2	1.671 (14)
O3—C3	1.152 (4)	C24A—Cl1A	1.794 (17)
O4—C4	1.147 (4)	C24A—H24C	0.9700
O5—C5	1.155 (4)	C24A—H24D	0.9700
O6—C6	1.148 (4)	C25—Cl3	1.741 (9)
O7—C7	1.149 (4)	C25—Cl3A	1.75 (2)
O8—C8	1.139 (4)	C25—Cl4	1.750 (4)
O9—C9	1.139 (4)	C25—H25A	0.9700
O10—C10	1.147 (4)	C25—H25B	0.9700
C11—C12	1.489 (6)		
			
C4—Fe1—C2	91.51 (17)	C12—C11—H11A	108.4
C4—Fe1—C1	99.37 (15)	S1—C11—H11A	108.4
C2—Fe1—C1	97.89 (17)	C12—C11—H11B	108.4
C4—Fe1—S2	85.96 (13)	S1—C11—H11B	108.4
C2—Fe1—S2	159.36 (12)	H11A—C11—H11B	107.4
C1—Fe1—S2	102.74 (12)	C11—C12—C13	114.2 (3)
C4—Fe1—S1	151.33 (11)	C11—C12—H12A	108.7
C2—Fe1—S1	87.50 (12)	C13—C12—H12A	108.7
C1—Fe1—S1	109.15 (11)	C11—C12—H12B	108.7
S2—Fe1—S1	85.04 (4)	C13—C12—H12B	108.7
C4—Fe1—Fe2	96.33 (11)	H12A—C12—H12B	107.6
C2—Fe1—Fe2	103.85 (12)	C12—C13—S2	116.7 (3)
C1—Fe1—Fe2	152.74 (12)	C12—C13—H13A	108.1
S2—Fe1—Fe2	56.24 (3)	S2—C13—H13A	108.1
S1—Fe1—Fe2	56.38 (3)	C12—C13—H13B	108.1
C5—Fe2—C3	90.13 (18)	S2—C13—H13B	108.1
C5—Fe2—S2	157.67 (12)	H13A—C13—H13B	107.3
C3—Fe2—S2	89.49 (12)	C15—C14—S4	118.8 (3)
C5—Fe2—S1	86.92 (13)	C15—C14—H14A	107.6
C3—Fe2—S1	157.92 (11)	S4—C14—H14A	107.6
S2—Fe2—S1	85.10 (4)	C15—C14—H14B	107.6
C5—Fe2—S5	106.91 (11)	S4—C14—H14B	107.6
C3—Fe2—S5	102.64 (11)	H14A—C14—H14B	107.0
S2—Fe2—S5	94.94 (3)	C16—C15—C14	121.5 (4)
S1—Fe2—S5	99.15 (3)	C16—C15—H15A	107.0
C5—Fe2—Fe1	102.21 (12)	C14—C15—H15A	107.0
C3—Fe2—Fe1	103.06 (10)	C16—C15—H15B	107.0
S2—Fe2—Fe1	56.27 (3)	C14—C15—H15B	107.0
S1—Fe2—Fe1	56.46 (3)	H15A—C15—H15B	106.7
S5—Fe2—Fe1	140.73 (3)	C15—C16—S3	118.0 (3)
C6—Fe3—C7	87.86 (16)	C15—C16—H16A	107.8
C6—Fe3—S4	89.82 (12)	S3—C16—H16A	107.8
C7—Fe3—S4	161.61 (12)	C15—C16—H16B	107.8
C6—Fe3—S3	152.86 (12)	S3—C16—H16B	107.8
C7—Fe3—S3	88.74 (11)	H16A—C16—H16B	107.1
S4—Fe3—S3	85.04 (3)	C18—C17—S6	112.5 (2)
C6—Fe3—S6	106.05 (12)	C18—C17—H17A	109.1
C7—Fe3—S6	97.86 (11)	S6—C17—H17A	109.1
S4—Fe3—S6	100.31 (3)	C18—C17—H17B	109.1
S3—Fe3—S6	101.09 (3)	S6—C17—H17B	109.1
C6—Fe3—Fe4	99.19 (12)	H17A—C17—H17B	107.8
C7—Fe3—Fe4	106.02 (11)	C17—C18—S7	111.7 (2)
S4—Fe3—Fe4	56.41 (3)	C17—C18—H18A	109.3
S3—Fe3—Fe4	56.14 (3)	S7—C18—H18A	109.3
S6—Fe3—Fe4	145.71 (3)	C17—C18—H18B	109.3
C10—Fe4—C8	93.52 (16)	S7—C18—H18B	109.3
C10—Fe4—C9	100.99 (17)	H18A—C18—H18B	107.9
C8—Fe4—C9	98.17 (16)	C20—C19—S7	110.6 (2)
C10—Fe4—S3	156.81 (13)	C20—C19—H19A	109.5
C8—Fe4—S3	86.09 (12)	S7—C19—H19A	109.5
C9—Fe4—S3	102.02 (12)	C20—C19—H19B	109.5
C10—Fe4—S4	86.56 (11)	S7—C19—H19B	109.5
C8—Fe4—S4	158.66 (12)	H19A—C19—H19B	108.1
C9—Fe4—S4	102.77 (12)	C19—C20—C21	116.8 (3)
S3—Fe4—S4	85.57 (3)	C19—C20—H20A	108.1
C10—Fe4—Fe3	100.97 (12)	C21—C20—H20A	108.1
C8—Fe4—Fe3	102.60 (12)	C19—C20—H20B	108.1
C9—Fe4—Fe3	148.60 (12)	C21—C20—H20B	108.1
S3—Fe4—Fe3	56.78 (3)	H20A—C20—H20B	107.3
S4—Fe4—Fe3	56.64 (3)	C20—C21—S8	111.9 (2)
S6—Ni1—S5	88.17 (3)	C20—C21—H21A	109.2
S6—Ni1—S8	174.85 (4)	S8—C21—H21A	109.2
S5—Ni1—S8	92.01 (3)	C20—C21—H21B	109.2
S6—Ni1—S7	91.05 (3)	S8—C21—H21B	109.2
S5—Ni1—S7	175.01 (4)	H21A—C21—H21B	107.9
S8—Ni1—S7	89.22 (3)	C23—C22—S8	108.5 (2)
C11—S1—Fe2	112.33 (15)	C23—C22—H22A	110.0
C11—S1—Fe1	113.21 (15)	S8—C22—H22A	110.0
Fe2—S1—Fe1	67.16 (3)	C23—C22—H22B	110.0
C13—S2—Fe2	111.11 (14)	S8—C22—H22B	110.0
C13—S2—Fe1	112.09 (15)	H22A—C22—H22B	108.4
Fe2—S2—Fe1	67.49 (3)	C22—C23—S5	111.9 (2)
C16—S3—Fe4	108.98 (14)	C22—C23—H23A	109.2
C16—S3—Fe3	115.56 (13)	S5—C23—H23A	109.2
Fe4—S3—Fe3	67.07 (3)	C22—C23—H23B	109.2
C14—S4—Fe4	110.26 (13)	S5—C23—H23B	109.2
C14—S4—Fe3	114.30 (13)	H23A—C23—H23B	107.9
Fe4—S4—Fe3	66.96 (3)	Cl1—C24—Cl2	116.0 (6)
C23—S5—Ni1	101.59 (11)	Cl1—C24—H24A	108.3
C23—S5—Fe2	110.57 (13)	Cl2—C24—H24A	108.3
Ni1—S5—Fe2	103.94 (3)	Cl1—C24—H24B	108.3
C17—S6—Ni1	99.83 (11)	Cl2—C24—H24B	108.3
C17—S6—Fe3	111.08 (12)	H24A—C24—H24B	107.4
Ni1—S6—Fe3	103.81 (3)	Cl2—C24A—Cl1A	115.0 (8)
C18—S7—C19	102.54 (16)	Cl2—C24A—H24C	108.5
C18—S7—Ni1	105.10 (11)	Cl1A—C24A—H24C	108.5
C19—S7—Ni1	103.26 (12)	Cl2—C24A—H24D	108.5
C22—S8—C21	101.18 (17)	Cl1A—C24A—H24D	108.5
C22—S8—Ni1	104.79 (11)	H24C—C24A—H24D	107.5
C21—S8—Ni1	103.69 (11)	C24A—Cl2—C24	29.9 (6)
O1—C1—Fe1	177.2 (3)	Cl3—C25—Cl3A	17.1 (14)
O2—C2—Fe1	178.3 (4)	Cl3—C25—Cl4	113.1 (3)
O3—C3—Fe2	174.9 (3)	Cl3A—C25—Cl4	105.2 (17)
O4—C4—Fe1	177.4 (3)	Cl3—C25—H25A	109.0
O5—C5—Fe2	175.9 (3)	Cl3A—C25—H25A	98.9
O6—C6—Fe3	174.5 (3)	Cl4—C25—H25A	109.0
O7—C7—Fe3	177.9 (3)	Cl3—C25—H25B	109.0
O8—C8—Fe4	177.5 (3)	Cl3A—C25—H25B	126.0
O9—C9—Fe4	178.7 (4)	Cl4—C25—H25B	109.0
O10—C10—Fe4	178.1 (4)	H25A—C25—H25B	107.8
C12—C11—S1	115.6 (3)		
			
C4—Fe1—Fe2—C5	−92.64 (18)	S6—Ni1—S5—Fe2	87.40 (4)
C2—Fe1—Fe2—C5	0.50 (18)	S8—Ni1—S5—Fe2	−97.75 (4)
C1—Fe1—Fe2—C5	142.4 (3)	S7—Ni1—S5—Fe2	6.4 (4)
S2—Fe1—Fe2—C5	−173.46 (13)	C5—Fe2—S5—C23	−16.11 (18)
S1—Fe1—Fe2—C5	77.91 (13)	C3—Fe2—S5—C23	−110.16 (17)
C4—Fe1—Fe2—C3	0.38 (18)	S2—Fe2—S5—C23	159.25 (12)
C2—Fe1—Fe2—C3	93.52 (18)	S1—Fe2—S5—C23	73.44 (12)
C1—Fe1—Fe2—C3	−124.6 (3)	Fe1—Fe2—S5—C23	120.04 (12)
S2—Fe1—Fe2—C3	−80.44 (13)	C5—Fe2—S5—Ni1	92.21 (14)
S1—Fe1—Fe2—C3	170.93 (13)	C3—Fe2—S5—Ni1	−1.84 (13)
C4—Fe1—Fe2—S2	80.83 (13)	S2—Fe2—S5—Ni1	−92.43 (4)
C2—Fe1—Fe2—S2	173.96 (13)	S1—Fe2—S5—Ni1	−178.24 (4)
C1—Fe1—Fe2—S2	−44.1 (2)	Fe1—Fe2—S5—Ni1	−131.64 (4)
S1—Fe1—Fe2—S2	−108.63 (4)	S5—Ni1—S6—C17	−144.38 (12)
C4—Fe1—Fe2—S1	−170.55 (13)	S8—Ni1—S6—C17	123.6 (4)
C2—Fe1—Fe2—S1	−77.41 (13)	S7—Ni1—S6—C17	30.69 (12)
C1—Fe1—Fe2—S1	64.5 (2)	S5—Ni1—S6—Fe3	100.88 (4)
S2—Fe1—Fe2—S1	108.63 (4)	S8—Ni1—S6—Fe3	8.9 (4)
C4—Fe1—Fe2—S5	130.07 (13)	S7—Ni1—S6—Fe3	−84.05 (4)
C2—Fe1—Fe2—S5	−136.80 (13)	C6—Fe3—S6—C17	−45.78 (17)
C1—Fe1—Fe2—S5	5.1 (2)	C7—Fe3—S6—C17	−135.81 (16)
S2—Fe1—Fe2—S5	49.24 (5)	S4—Fe3—S6—C17	47.03 (12)
S1—Fe1—Fe2—S5	−59.39 (5)	S3—Fe3—S6—C17	133.94 (12)
C6—Fe3—Fe4—C10	5.20 (16)	Fe4—Fe3—S6—C17	90.02 (13)
C7—Fe3—Fe4—C10	95.60 (16)	C6—Fe3—S6—Ni1	60.67 (13)
S4—Fe3—Fe4—C10	−78.42 (12)	C7—Fe3—S6—Ni1	−29.36 (12)
S3—Fe3—Fe4—C10	172.88 (12)	S4—Fe3—S6—Ni1	153.48 (3)
S6—Fe3—Fe4—C10	−132.06 (12)	S3—Fe3—S6—Ni1	−119.61 (4)
C6—Fe3—Fe4—C8	−90.97 (16)	Fe4—Fe3—S6—Ni1	−163.53 (4)
C7—Fe3—Fe4—C8	−0.57 (16)	S6—Ni1—S7—C18	−12.91 (13)
S4—Fe3—Fe4—C8	−174.59 (11)	S5—Ni1—S7—C18	68.0 (4)
S3—Fe3—Fe4—C8	76.71 (11)	S8—Ni1—S7—C18	172.23 (13)
S6—Fe3—Fe4—C8	131.77 (12)	S6—Ni1—S7—C19	−120.04 (12)
C6—Fe3—Fe4—C9	138.8 (3)	S5—Ni1—S7—C19	−39.2 (5)
C7—Fe3—Fe4—C9	−130.8 (3)	S8—Ni1—S7—C19	65.10 (12)
S4—Fe3—Fe4—C9	55.2 (2)	S6—Ni1—S8—C22	97.6 (4)
S3—Fe3—Fe4—C9	−53.5 (2)	S5—Ni1—S8—C22	5.75 (13)
S6—Fe3—Fe4—C9	1.6 (2)	S7—Ni1—S8—C22	−169.41 (13)
C6—Fe3—Fe4—S3	−167.68 (12)	S6—Ni1—S8—C21	−156.7 (4)
C7—Fe3—Fe4—S3	−77.28 (12)	S5—Ni1—S8—C21	111.45 (12)
S4—Fe3—Fe4—S3	108.70 (4)	S7—Ni1—S8—C21	−63.71 (12)
S6—Fe3—Fe4—S3	55.06 (6)	C4—Fe1—C1—O1	15 (7)
C6—Fe3—Fe4—S4	83.62 (12)	C2—Fe1—C1—O1	−78 (7)
C7—Fe3—Fe4—S4	174.02 (12)	S2—Fe1—C1—O1	103 (7)
S3—Fe3—Fe4—S4	−108.70 (4)	S1—Fe1—C1—O1	−168 (7)
S6—Fe3—Fe4—S4	−53.64 (6)	Fe2—Fe1—C1—O1	139 (7)
C5—Fe2—S1—C11	146.3 (2)	C4—Fe1—C2—O2	−3 (13)
C3—Fe2—S1—C11	−130.9 (4)	C1—Fe1—C2—O2	97 (13)
S2—Fe2—S1—C11	−54.54 (16)	S2—Fe1—C2—O2	−85 (13)
S5—Fe2—S1—C11	39.69 (16)	S1—Fe1—C2—O2	−154 (13)
Fe1—Fe2—S1—C11	−106.82 (16)	Fe2—Fe1—C2—O2	−99 (13)
C5—Fe2—S1—Fe1	−106.85 (11)	C5—Fe2—C3—O3	71 (4)
C3—Fe2—S1—Fe1	−24.1 (3)	S2—Fe2—C3—O3	−87 (4)
S2—Fe2—S1—Fe1	52.28 (3)	S1—Fe2—C3—O3	−12 (4)
S5—Fe2—S1—Fe1	146.51 (3)	S5—Fe2—C3—O3	178 (100)
C4—Fe1—S1—C11	125.4 (3)	Fe1—Fe2—C3—O3	−32 (4)
C2—Fe1—S1—C11	−146.0 (2)	C2—Fe1—C4—O4	−90 (9)
C1—Fe1—S1—C11	−48.5 (2)	C1—Fe1—C4—O4	172 (8)
S2—Fe1—S1—C11	53.28 (16)	S2—Fe1—C4—O4	70 (9)
Fe2—Fe1—S1—C11	105.54 (16)	S1—Fe1—C4—O4	−2 (9)
C4—Fe1—S1—Fe2	19.9 (3)	Fe2—Fe1—C4—O4	14 (9)
C2—Fe1—S1—Fe2	108.47 (12)	C3—Fe2—C5—O5	−88 (5)
C1—Fe1—S1—Fe2	−154.04 (13)	S2—Fe2—C5—O5	1 (6)
S2—Fe1—S1—Fe2	−52.26 (3)	S1—Fe2—C5—O5	70 (5)
C5—Fe2—S2—C13	123.1 (4)	S5—Fe2—C5—O5	169 (5)
C3—Fe2—S2—C13	−147.83 (19)	Fe1—Fe2—C5—O5	16 (5)
S1—Fe2—S2—C13	53.60 (16)	C7—Fe3—C6—O6	−60 (4)
S5—Fe2—S2—C13	−45.19 (16)	S4—Fe3—C6—O6	102 (4)
Fe1—Fe2—S2—C13	106.04 (16)	S3—Fe3—C6—O6	23 (4)
C5—Fe2—S2—Fe1	17.0 (3)	S6—Fe3—C6—O6	−157 (4)
C3—Fe2—S2—Fe1	106.13 (11)	Fe4—Fe3—C6—O6	46 (4)
S1—Fe2—S2—Fe1	−52.44 (3)	C6—Fe3—C7—O7	34 (9)
S5—Fe2—S2—Fe1	−151.23 (3)	S4—Fe3—C7—O7	−49 (9)
C4—Fe1—S2—C13	155.00 (18)	S3—Fe3—C7—O7	−119 (9)
C2—Fe1—S2—C13	−121.5 (4)	S6—Fe3—C7—O7	140 (9)
C1—Fe1—S2—C13	56.29 (17)	Fe4—Fe3—C7—O7	−65 (9)
S1—Fe1—S2—C13	−52.24 (14)	C10—Fe4—C8—O8	176 (100)
Fe2—Fe1—S2—C13	−104.63 (14)	C9—Fe4—C8—O8	75 (8)
C4—Fe1—S2—Fe2	−100.38 (11)	S3—Fe4—C8—O8	−27 (8)
C2—Fe1—S2—Fe2	−16.8 (4)	S4—Fe4—C8—O8	−94 (8)
C1—Fe1—S2—Fe2	160.92 (11)	Fe3—Fe4—C8—O8	−82 (8)
S1—Fe1—S2—Fe2	52.38 (3)	C10—Fe4—C9—O9	−122 (17)
C10—Fe4—S3—C16	−128.5 (3)	C8—Fe4—C9—O9	−27 (17)
C8—Fe4—S3—C16	141.69 (18)	S3—Fe4—C9—O9	61 (17)
C9—Fe4—S3—C16	44.16 (18)	S4—Fe4—C9—O9	149 (17)
S4—Fe4—S3—C16	−57.98 (14)	Fe3—Fe4—C9—O9	105 (17)
Fe3—Fe4—S3—C16	−110.49 (14)	C8—Fe4—C10—O10	96 (10)
C10—Fe4—S3—Fe3	−18.0 (3)	C9—Fe4—C10—O10	−165 (10)
C8—Fe4—S3—Fe3	−107.82 (11)	S3—Fe4—C10—O10	8 (10)
C9—Fe4—S3—Fe3	154.65 (12)	S4—Fe4—C10—O10	−63 (10)
S4—Fe4—S3—Fe3	52.51 (3)	Fe3—Fe4—C10—O10	−8 (10)
C6—Fe3—S3—C16	128.4 (3)	Fe2—S1—C11—C12	66.6 (3)
C7—Fe3—S3—C16	−148.79 (19)	Fe1—S1—C11—C12	−7.1 (4)
S4—Fe3—S3—C16	48.53 (16)	S1—C11—C12—C13	−63.0 (4)
S6—Fe3—S3—C16	−51.03 (16)	C11—C12—C13—S2	63.8 (4)
Fe4—Fe3—S3—C16	100.90 (16)	Fe2—S2—C13—C12	−66.6 (3)
C6—Fe3—S3—Fe4	27.5 (3)	Fe1—S2—C13—C12	6.8 (3)
C7—Fe3—S3—Fe4	110.31 (11)	Fe4—S4—C14—C15	−58.3 (4)
S4—Fe3—S3—Fe4	−52.37 (3)	Fe3—S4—C14—C15	14.7 (4)
S6—Fe3—S3—Fe4	−151.92 (3)	S4—C14—C15—C16	49.9 (6)
C10—Fe4—S4—C14	−145.83 (19)	C14—C15—C16—S3	−53.0 (6)
C8—Fe4—S4—C14	123.3 (3)	Fe4—S3—C16—C15	64.1 (4)
C9—Fe4—S4—C14	−45.33 (18)	Fe3—S3—C16—C15	−8.9 (4)
S3—Fe4—S4—C14	56.01 (14)	Ni1—S6—C17—C18	−48.1 (3)
Fe3—Fe4—S4—C14	108.64 (14)	Fe3—S6—C17—C18	60.9 (3)
C10—Fe4—S4—Fe3	105.53 (12)	S6—C17—C18—S7	41.3 (3)
C8—Fe4—S4—Fe3	14.6 (3)	C19—S7—C18—C17	94.0 (3)
C9—Fe4—S4—Fe3	−153.97 (12)	Ni1—S7—C18—C17	−13.7 (3)
S3—Fe4—S4—Fe3	−52.63 (3)	C18—S7—C19—C20	179.8 (2)
C6—Fe3—S4—C14	156.07 (18)	Ni1—S7—C19—C20	−71.2 (2)
C7—Fe3—S4—C14	−121.3 (4)	S7—C19—C20—C21	69.7 (4)
S3—Fe3—S4—C14	−50.61 (14)	C19—C20—C21—S8	−68.6 (4)
S6—Fe3—S4—C14	49.78 (15)	C22—S8—C21—C20	177.2 (2)
Fe4—Fe3—S4—C14	−102.76 (14)	Ni1—S8—C21—C20	68.8 (2)
C6—Fe3—S4—Fe4	−101.17 (12)	C21—S8—C22—C23	−140.8 (2)
C7—Fe3—S4—Fe4	−18.5 (4)	Ni1—S8—C22—C23	−33.2 (2)
S3—Fe3—S4—Fe4	52.14 (3)	S8—C22—C23—S5	51.1 (3)
S6—Fe3—S4—Fe4	152.54 (3)	Ni1—S5—C23—C22	−43.7 (3)
S6—Ni1—S5—C23	−157.74 (13)	Fe2—S5—C23—C22	66.2 (3)
S8—Ni1—S5—C23	17.11 (13)	Cl1A—C24A—Cl2—C24	77.7 (16)
S7—Ni1—S5—C23	121.3 (4)	Cl1—C24—Cl2—C24A	−74.2 (14)

### Hydrogen-bond geometry (Å, º)

**Table:**

*D*—H···*A*	*D*—H	H···*A*	*D*···*A*	*D*—H···*A*
C19—H19*A*···O3	0.97	2.52	3.440 (5)	159
C15—H15*B*···S6	0.97	2.83	3.578 (4)	134
C13—H13*B*···O4^i^	0.97	2.57	3.470 (5)	154
C20—H20*A*···O8^ii^	0.97	2.57	3.263 (5)	128
C24—H24*A*···O7^iii^	0.97	2.44	3.08 (2)	123
C24*A*—H24*C*···O7^iii^	0.97	2.42	3.32 (2)	154

Symmetry codes: (i) −*x*+1, −*y*+2, −*z*+1; (ii) −*x*+2, −*y*+1, −*z*; (iii) *x*−1, *y*, *z*+1.
